# FXII decline during cardiopulmonary bypass and the limits of routine coagulation assays: a prospective observational analysis

**DOI:** 10.1186/s13741-026-00687-0

**Published:** 2026-04-30

**Authors:** Sarah Thaler, Isabell Aster, Emmanuelle Patton, Charlotte Voigt, Sven Peterss, Matthias Feuerecker, Philipp Groene

**Affiliations:** 1 Department of Anaesthesiology, LMU University Hospital, LMU Munich, Marchioninistr. 15, Munich, 81377 Germany; 2 Department of Cardiac Surgery, LMU University Hospital, LMU Munich, Marchioninistr. 15, Munich, 81377 Germany

**Keywords:** Cardiopulmonary bypass (CPB), Intrinsic coagulation, Coagulation factor XII (FXII), Coagulation factor VIII (FVIII), Coagulation factor V (FV), aPTT, Viscoelastometry

## Abstract

**Background:**

The contact activation system is triggered when blood comes into contact with artificial surfaces. Cardiopulmonary bypass (CPB) induces such activation, however the extent of factor XII (FXII) reduction and its reflection in routine coagulation assays remain unclear.

**Methods:**

In this prospective observational study, 20 adult patients undergoing elective cardiac surgery with CPB (10 coronary artery bypass grafting (CABG), 10 aortic aneurysm repair) were analyzed. Blood samples were collected immediately prior to anesthesia induction (T1) and three minutes after protamine administration (T2). Laboratory analyses included FXII, factor VIII (FVIII), and factor V (FV) activities, activated partial thromboplastin time (aPTT), prothrombin time (Quick/INR), fibrinogen, and viscoelastic testing (ROTEM).

**Results:**

FXII activity decreased significantly after CPB (whole cohort: 98% (79/122) vs. 62% (51/83); CABG: 108% (83/124) vs. 64% (47/85); aortic: 92% (75/119) vs. 61% (52/82), *p* < 0.001 each). FV declined, whereas FVIII tended to increase. aPTT prolongation was minimal and remained within the normal range. A strong correlation between FXII decline and aPTT change was observed only in CABG patients (ρ = − 0.835, *p* = 0.003). ROTEM clotting times were moderately prolonged after CPB but showed no correlation with FXII activity.

**Conclusions:**

CPB induced a significant reduction in FXII activity. Global coagulation assays such as aPTT or ROTEM have limited sensitivity for contact factor deficiencies. Isolated prolongation of these parameters after CPB should be interpreted cautiously and, in the absence of clinical bleeding, should not routinely prompt hemostatic interventions. Future studies should address subgroup differences (CABG vs. aortic surgery) in larger cohorts.

**Trial registration:**

The trial was registered in the German Clinical Trials Register (DRKS00034174).

## Background

The contact activation system, consisting of factor XII (FXII), prekallikrein, and high-molecular-weight kininogen, represents the initiation mechanism of the intrinsic coagulation pathway once blood is exposed to negatively charged foreign surfaces (Renné et al. 2012, Veen et al. 2009). Although FXII is part of the intrinsic pathway, its deficiency is not associated with a bleeding tendency, underscoring its limited role in physiological hemostasis. Instead, FXII and its related proteins exert diverse biological functions, including anticoagulant, profibrinolytic, and proinflammatory effects through interactions with endothelial cells, platelets, and leukocytes (Maas and Renné 2018, Schmaier and McCrae 2007, Long et al. 2016).

Cardiac surgery requiring cardiopulmonary bypass (CPB) exposes circulating blood to extensive artificial surfaces and non-physiological flow conditions. This surface contact triggers activation of the contact system leading to widespread alterations in coagulation and inflammation (Veen et al. 2009, Paparella et al. 2004, Besser and Klein 2010). To counteract the resulting procoagulant state, high-dose heparinization is required, which in turn necessitates careful intraoperative and postoperative anticoagulant management. Following CPB, a transient coagulopathy develops due to factor consumption, hemodilution, platelet dysfunction, and inflammatory activation (Grottke et al. 2015). Residual heparin as well as protamine overdose may also contribute to post-CPB coagulopathy and prolongation of clotting times in both conventional coagulation assays and viscoelastic tests (Activated Clotting Time (ACT), activated Partial Thromboplastin Time (aPTT, INTEM/HEPTEM CT) (Ichikawa et al. 2014, Ni Ainle et al. 2009, Ortmann et al. 2013, Meesters et al. 2016).

The activated partial thromboplastin time (aPTT) is a commonly used laboratory assay reflecting the integrity of the intrinsic and common coagulation pathway and serves as a monitoring tool for unfractionated heparin therapy. However, the aPTT is influenced by numerous analytical and pre-analytical variables, and its sensitivity to individual coagulation factors, including FXII, varies depending on the reagent used (Veen et al. 2009, Kitchen 2000). Both insufficient and excessive sensitivity of the aPTT to FXII deficiency may impair its clinical reliability. Clinically, prolonged aPTT and ACT after CPB are commonly interpreted as indicators of residual heparin effects and increased bleeding risk. However, prolonged aPTT and ACT after protamine administration due to acquired FXII deficiency, protamine overdose, or Lupus anticoagulants may lead to misinterpretation of these results. Knowledge about the interference and limitations of the assays used in each institution are essential for correct result interpretation to avoid misclassification of bleeding risks (Kitchens 2005, Dembitzer et al. 2010). Conversely, a reduction in FXII activity may not necessarily manifest as a prolonged aPTT, potentially limiting the test’s reliability after CPB.

Despite the well-known activation of the contact system during extracorporeal circulation, data on specific FXII activity changes during and after CPB are limited. Moreover, the relationship between such changes and routinely used coagulation assays has not been clearly established. Understanding this relationship is clinically relevant because misinterpretation of standard coagulation tests may affect postoperative anticoagulation management and diagnostic decisions. Therefore, in this prospective cohort study of patients undergoing cardiac surgery with CPB, we investigated whether FXII activity decreased during CPB and whether this change is reflected by conventional coagulation assays such as the aPTT.

## Methods

### Study design and population

This prospective observational study included adult patients undergoing elective cardiac surgery requiring CPB at LMU University Hospital Munich. Patients were assigned to one of two predefined surgical groups: coronary artery bypass grafting (CABG) or elective aortic aneurysm repair. These procedures were chosen because they represent major cardiac operations that differ in duration, complexity, and perfusion techniques. Aortic surgery was routinely performed under hypothermic circulatory arrest at 25 °C.

The study protocol was approved by the Ethics Committee of LMU Munich, Germany (No 24–0231; 2024, April 17) and the study was conducted in accordance with institutional guidelines and the Declaration of Helsinki. The trial was registered in the German Clinical Trials Register (DRKS00034174). Written informed consent was obtained from all participants prior to inclusion.

Exclusion criteria were age < 18 years, refusal to participate, congenital or acquired coagulation disorders (based on medical history or coagulation questionnaire), and the use of anticoagulant medication within 12 h before surgery, except for antiplatelet agents.

### Surgical procedure and anticoagulant management

CPB was established using standard institutional techniques. Heparin was administered at a dose of 400 IU/kg body weight, supplemented with additional 10.000 IU added to the CPB circuit. After termination of CPB, protamine was administered in a 1:1 ratio to the total initial heparin dose (400IU/kg plus 10.000 IU in the CPB circuit) via central venous catheter using an infusion pump over approximately 10 min, in accordance with institutional standards.

### Blood sampling

Blood samples were collected at two time points:

- T1 – Immediately prior to induction of general anesthesia (baseline), and
- T2 – Three minutes after protamine administration following the cessation of CPB and before administration of coagulation products or blood transfusions.

Samples were drawn from an indwelling arterial catheter and immediately processed for laboratory and viscoelastic analyses. Laboratory parameters included the activity of coagulation factors XII, VIII, and V, as well as aPTT (Dade^®^ Actin^®^ FSL), prothrombin time (Quick/INR), platelet count, and fibrinogen concentration. Analyses were performed by the Department of Laboratory Medicine at LMU Munich as part of standard diagnostic testing.

Viscoelastic testing was performed using a ROTEM sigma analyzer (Tem Innovations GmbH, Munich, Germany) with the assays INTEM, HEPTEM, EXTEM, and FIBTEM. INTEM assesses the intrinsic pathway using ellagic acid as an activator, HEPTEM differentiates heparin effects through the addition of heparinase, EXTEM evaluates the extrinsic pathway via tissue factor activation, and FIBTEM isolates fibrin polymerization by inhibiting platelets with cytochalasin D and tirofiban. EXTEM and FIBTEM neutralize heparin up to 5 IU/ml by polybrene; HEPTEM neutralizes heparin up to 7 IU/ml by Heparinase. All tests were conducted at 37 °C in accordance with manufacturer’s instructions.

The following ROTEM variables were recorded: clotting time (CT), clot formation time (CFT), amplitudes after 5 and 20 min (A5, A20), and maximum clot firmness (MCF).

### Statistical analysis

Preliminary internal measurements of FXII activity changes during CPB indicated a large effect size (Cohen’s d = 1.29). Based on this assumption, a priori power analysis determined that a minimum of 12 patients would be required to achieve a power of 95% (alpha = 0.05). To account for potential dropouts, 20 patients were included.

Data from all patients were analyzed as a single cohort to assess the overall effect of CPB on FXII activity and coagulation parameters. Subgroup analyses for CABG and aortic surgery were performed to evaluate potential procedure-related differences.

Data distribution was assessed visually and by the Shapiro-Wilk test. Paired t-tests were used for normally distributed data, and Wilcoxon-Mann-Whitney U test otherwise. Correlations were analyzed using Pearson’s or Spearman’s method, as appropriate. Results are presented as median with interquartile range (IQR) unless stated otherwise. Statistical analyses were performed using IBM SPSS Statistics, version 29.0.0 (IBM Corp., Armonk, NY, USA). A two-sided p-value < 0.05 was considered statistically significant.

## Results

### Patient characteristics

Between June 2024 and November 2024, 22 patients scheduled for elective cardiac surgery using CPB were screened for inclusion. Two were excluded due to incomplete data, leaving 20 patients for final analysis. Ten patients underwent ascending aortic aneurysm repair with moderate hypothermic circulatory arrest at 25 °C, and ten underwent normothermic coronary artery bypass grafting (CABG). Baseline patient characteristics and intraoperative variables, including type and duration of surgery, CPB time, blood loss, transfusion volumes, and coagulation product use, are summarized in Table 1.

**Table 1:** Patient characteristics and surgery data

Parameter	Whole cohort (*n* = 20)	CABG cohort (*n* = 10)	Aortic cohort (*n* = 10)
	Patient characteristics		
Age (years)	64 (61/70)	64 (63/71)	66 (59/71)
Sex (male/female)	13 (65%)/7 (35%)	6 (60%)/4 (40%)	7 (70%)/3 (30%)
BMI (kg/m^2^)	26 (22/30)	26 (21/30)	27 (24/29)
	Surgery data		
Length of surgery (min)	411 (264/463)	360 (217/450)	425 (342/494)
CPB time (min)	202 (112/260)	127 (77/184)	256 (233/305)
Aortic cross clamp time (min)	149 (72/197)	88 (62/138)	189 (162/238)
Hypothermic circulatory arrest time (min)			22 (14/30)
	Intraoperative volume turnover		
Cristalloids (ml)	3436 (2632/3916)	3633 (2389/4084)	3154 (2689/3631)
Colloids (ml)	0 (0/75)	0 (0/0)	0 (0/200)
Blood loss (ml)	1000 (725/2300)	775 (500/1500)	1950 (800/2625)
Urine output (ml)	1451 (1016/3475)	1100 (908/2388)	2450 (1266/5300)
	Transfusion and coagulation substitution		
Red Blood Cell concentrate (ml)	0 (0/250)	0 (0/250)	0 (0/63)
Autologous blood transfusion (ml)	325 (22/686)	193 (0/450)	456 (260/893)
Platelet concentrate (ml)	0 (0/225)	0 (0/0)	0 (0/300)
Fresh Frozen Plasma (ml)	375 (0/938)	0 (0/750)	750 (0/1500)
Fibrinogen (g)	3 (2/3)	2 (1.5/3)	3 (2.75/4)
Prothrombin complex concentrate (I.E.)	3000 (1500/3900)	1750 (750/2325)	3800 (3000/4800)

Data are presented as median with IQR or percentage, as appropriate. The table summarizes baseline demographics, surgical characteristics, intraoperative fluid turnover, and transfusion or coagulation product use in the total study population (*n* = 20) and in the two predefined subgroups undergoing CABG (*n* = 10) and aortic aneurysm repair (*n* = 10)

*BMI* Body Mass Index, *CABG* Coronary Artery Bypass Grafting, *CPB* Cardiopulmonary Bypass

### FXII activity

At baseline, all patients showed normal FXII activity (whole cohort 98% (IQR 79/122); CABG 108% (83/124); aortic 92% (75/119)).

**Fig. 1:**
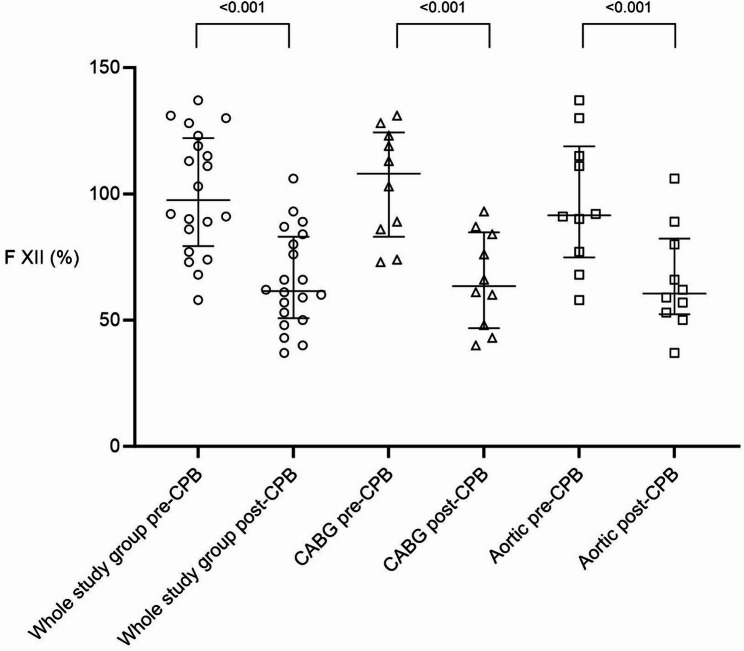
Factor XII (FXII) activity before and after cardiopulmonary bypass (CPB) in the overall cohort and surgical subgroups. Figure 1: FXII activity (%) in the whole study population and in CABG and aortic surgery subgroups before and after CPB. Each data point represents an individual patient; horizontal lines indicate median and IQR. The decreases in FXII activity post-CPB were statistically significant throughout all study groups. CABG: Coronary Artery Bypass Grafting; CPB: Cardiopulmonary Bypass; FXII: Factor XII

FXII activity decreased significantly after CPB in the total cohort as well as within both surgical subgroups (whole cohort 62% (51/83), *p* < 0.001; CABG 64% (47/85), *p* < 0.001; aortic 61% (52/82), *p* < 0.001). Despite this marked decline, FXII activity remained within or just below the lower reference limit in most patients. Post-CPB FXII values below 60% were observed in 8 out of 20 patients (40%), slightly more frequently after aortic surgery (5/10) than CABG (3/10). The distribution of FXII activity at both time points is depicted in Fig. 1.

### Other coagulation factors

Factor V activity decreased significantly after CPB in all cohorts, while FVIII activity showed a nonsignificant trend toward higher values. Median values for all measured coagulation factors before and after CPB are summarized in Table 2.

**Table 2:** Changes in coagulation factors before and after cardiopulmonary bypass (CPB)

	Whole cohort (*n* = 20)			CABG cohort (*n* = 10)			Aortic cohort (*n* = 10)		
	Pre-CPB (T1)	Post-CPB (T2)	*p*-value	Pre-CPB (T1)	Post-CPB (T2)	*p*-value	Pre-CPB (T1)	Post-CPB (T2)	*p*-value
FXII (%)	98 (79/122)	62 (51/83)	< 0.001	108 (83/124)	64 (47/85)	< 0.001	92 (75/119)	61 (52/82)	< 0.001
FV (%)	112 (89/126)	60 (54/68)	< 0.001	89 (81/132)	61 (55/77)	< 0.001	117 (104/129)	60 (53/65)	< 0.001
FVIII (%)	158 (129/207)	170 (141/247)	0.467	167 (119/232)	195 (137/271)	0.575	154 (131/178)	170 (131/221)	0.683

Values are expressed as median with IQR. The table summarizes plasma activities of FXII, FV, and FVIII before (T1) and after (T2) CPB in the whole cohort (*n* = 20) and in the two predefined subgroups. Statistical comparison between time points were performed using paired t-test or Wilcoxon signed-rank test, as appropriate

*CABG* Coronary Artery Bypass Grafting, *CPB* Cardiopulmonary Bypass, *FXII* Factor XII, *FV* Factor V, *FVIII* Factor VIII

### Standard coagulation tests

The aPTT was slightly prolonged after CPB but remained within the normal range (whole cohort 25 s vs. 28 s, *p* = 0.004; CABG 25 s vs. 27 s, *p* = 0.092; aortic 24 s vs. 28 s, *p* = 0.022).

Changes in Quick/INR and fibrinogen levels were observed after CPB; however, for these parameters only a descriptive statistical analysis was performed (data not shown).

### Viscoelastic testing

The intrinsically activated ROTEM assays (INTEM and HEPTEM) demonstrated a prolongation of clotting time (CT) after CPB in both subgroups. Detailed viscoelastic parameters for all assays and time points are shown in Table 3.

**Table 3:** ROTEM variables before and after CPB

		Whole study group		CABG cohort		Aortic cohort	
		Pre-CPB (T1)	Post-CPB (T2)	Pre-CPB (T1)	Post-CPB (T2)	Pre-CPB (T1)	Post-CPB (T2)
EXTEM	CT	61 (56/64)	76 (67/88)	63 (58/66)	74 (68/90)	61 (54/61)	78 (63/88)
	CFT	61 (56/73)	81 (70/91)	63 (57/80)	73 (62/84)	61 (51/74)	86 (78/92)
	A5	48 (45/50)	40 (39/45)	48 (42/50)	43 (39/46)	48 (45/51)	40 (39/40)
	A20	63 (60/65)	56 (54/60)	63 (58/65)	58 (55/62)	63 (61/66)	55 (54/56)
	MCF	63 (61/66)	57 (55/61)	64 (59/66)	60 (57/63)	63 (62/67)	56 (54/58)
FIBTEM	CT	59 (52/63)	76 (66/84)	56 (50/62)	75 (70/83)	59 (56/65)	78 (64/88)
	CFT	0 (0/0)	0 (0/0)	0 (0/14)	0 (0/0)	0 (0/0)	0 (0/0)
	A5	12 (10/14)	11 (9/13)	12 (10/16)	11 (9/14)	12 (10/13)	11 (8/13)
	A20	14 (11/17)	13 (10/16)	13 (11/19)	13 (10/17)	15 (11/16)	13 (9/15)
	MCF	14 (11/18)	13 (10/17)	13 (11/21)	13 (10/18)	16 (11/17)	13 (10/16)
INTEM	CT	188 (167/206)	302 (253/357)	194 (178/209)	275 (243/328)	176 (164/197)	334 (284/436)
	CFT	68 (59/80)	117 (101/137)	68 (62/91)	105 (92/124)	66 (58/78)	127 (111/145)
	A5	46 (43/49)	35 (33/38)	45 (40/49)	37 (33/39)	47 (44/50)	34 (32/35)
	A20	60 (58/64)	52 (49/54)	60 (55/64)	54 (50/56)	60 (59/65)	51 (48/53)
	MCF	60 (58/64)	52 (49/57)	60 (55/65)	55 (51/58)	61 (59/65)	52 (49/55)
HEPTEM	CT	193 (173/205)	303 (238/364)	194 (176/207)	282 (232/332)	178 (169/204)	336 (281/424)
	CFT	78 (66/89)	127 (103/150)	80 (65/98)	108 (100/138)	78 (66/85)	136 (123/158)
	A5	44 (40/46)	33 (31/37)	43 (39/46)	36 (32/38)	44 (41/46)	32 (30/33)
	A20	59 (56/62)	51 (47/54)	59 (54/61)	53 (50/55)	59 (56/62)	49 (45/51)
	MCF	59 (56/62)	52 (48/56)	59 (54/62)	55 (52/57)	59 (56/63)	50 (46/53)
INTEM/HEPTEM CT-ratio		188/193 = 0.97	302/303 = 1	194/194 = 1	275/282 = 0.98	176/178 = 0.99	334/336 = 0.99

Values are presented as median with IQR. The table displays viscoelastic parameters obtained from INTEM, HEPTEM, EXTEM, and FIBTEM assays at baseline before CPB (T1) and after cessation of CPB (T2) in the total cohort and surgical subgroups

*A5/A20* Amplitude after 5/20 minutes, *CABG* Coronary Artery Bypass Grafting, *CFT* Clot Formation Time, *CPB* Cardiopulmonary Bypass, *CT* Clotting Time, *MCF* Maximum Clot Firmness

### Correlation analyses

In CABG patients, the relative change in FXII activity during CPB showed a strong negative correlation with the corresponding change in aPTT (*ρ* = − 0.835, *p* = 0.003).

For the entire study population, this relationship displayed the same trend but did not reach statistical significance (*ρ* = − 0.427, *p* = 0.06).

No significant correlation was found between FXII activity and aPTT in the aortic surgery group (*ρ* = − 0.312, *p* = 0.38), nor between FXII activity and HEPTEM-CT in any subgroup (whole cohort *ρ = -0.089*, *p = 0.71*; CABG *ρ = 0.091*,* p = 0.802*; aortic *ρ = -0.164*,* p = 0.65)*.

FXII activity did not correlate with the duration of surgery (whole cohort *ρ = -0.109*,* p = 0.647*; CABG *ρ = -0.395*,* p = 0.258*; aortic *ρ = 0.036*,* p = 0.920*) or CPB time (whole cohort *ρ = 0.122*,* p = 0.608*; CABG *ρ = -0.188*,* p = 0.602*; aortic *ρ = -0.067*,* p = 0.854*) nor with intraoperative blood loss (whole cohort *ρ = 0.196*,* p = 0.407*; CABG *ρ = 0.089*,* p = 0.808*; aortic *ρ = 0.353*,* p = 0.317*) in the overall cohort or within the surgical subgroups.

## Discussion

### Principal findings

This study demonstrates that CPB leads to a significant decline in FXII and FV activity, whereas FVIII activity shows a tendency to increase. Median post-CPB FXII levels remained within or just below the lower reference range, hence the corresponding aPTT changes were minimal. Subgroup-specific differences were observed with a strong correlation between FXII decline and aPTT prolongation in the CABG cohort, whereas no such correlation was found in the aortic subgroup.

### Interpretation in the context of the aPTT

A reduction of FXII would generally be expected to prolong the aPTT; however, in this study, a post-CPB decline in FXII within the lower reference range was accompanied with only minor changes in aPTT. This finding may be attributed to the analytical properties of the aPTT assay. Clot time-based assays such as the aPTT primarily reflect the initiation phase of coagulation and capture only a small fraction of total thrombin generation. Therefore, alterations in early intrinsic pathway factors like FXII may not translate into changes in clotting times.

The aPTT is influenced by numerous pre-analytical and analytical variables, including tube type, sample handling, pharmacological agents like heparin and protamine, and the presence of antiphospholipid antibodies such as lupus anticoagulant (Veen et al. 2009, Kitchen 2000). Moreover, different reagents vary substantially in their sensitivity toward individual intrinsic pathway factors (Veen et al. 2009, Kitchen 2000). Previous studies have shown that the aPTT’s ability to detect FXII deficiency can be limited, with detection thresholds ranging from 16% to 41% (Marlar et al. 1984, Turi and Peerschke 1986, Toulon et al. 2016). Mild or even moderate FXII reductions may therefore remain undetected. Clinically, this implies that normal or only mildly prolonged aPTT values do not exclude relevant FXII depletion. In a critically ill cohort, an FXII activity threshold of 42.5% was identified as the point below which aPTT prolongation becomes apparent (Bachler et al. 2019). In the present study, median post-CPB FXII levels were approximately 60%, which may explain why no overt aPTT prolongation was observed. From a physiological perspective, the degree of FXII reduction found in this study is unlikely to be sufficient to impair hemostasis, given the well-established FXII-independent activation of factor XI by thrombin (Kravtsov et al. 2009, Oliver et al. 1999, Naito and Fujikawa 1991). Thus, the observed FXII decrease should be regarded as a laboratory and interpretative phenomenon rather than a mechanism contributing to postoperative bleeding. Additionally, FXII represents only one determinant of the aPTT, which is also affected by FV and FVIII. The simultaneous CPB-related decline in FV and the opposing increase in FVIII may have offset each other’s effects, thereby attenuating the overall impact of FXII reduction on the aPTT.

A previous study investigating contact pathway activation during CPB also reported a decline in FXII levels accompanied by increased FXIIa-like and kallikrein activities, indicating pronounced biochemical activation of the contact system (Wendel et al. 1999). In contrast to the predominantly chromogenic activity-based assays used in that study, the present work focused on functional clotting factor activities and global coagulation assays. This methodological difference might explain why, despite similar FXII depletion, our findings demonstrate only minimal effects on aPTT supporting the concept that CPB-induced FXII activation has limited impact on effective hemostasis.

### ROTEM findings

The interpretation of HEPTEM CT changes after CPB requires differentiation between the CABG and aortic cohort. While CT prolongation was moderate in the CABG group (approximately 45%), it was markedly more pronounced in the aortic cohort (approximately 90%), indicating a substantially greater disturbance of coagulation in the latter. However, these changes did not correlate with FXII activity (CABG: *ρ = 0.091; aortic: ρ = -0.164)*. This aligns with the concept that ROTEM primarily assesses the dynamics of thrombin generation and clot propagation rather than being sensitive to isolated deficiencies of contact factors (Drotarova et al. 2023). As FXII is dispensable for effective hemostasis in vivo, its reduction is unlikely to functionally influence clot initiation as measured by ROTEM. In addition, concomitant increase in FVIII may further counterbalance the effects of FXII and FV reductions on clot initiation. In this context, the INTEM/HEPTEM CT ratio may provide additional information to differentiate between residual heparin effect and coagulation factor deficiency or inhibition (Ichikawa et al. 2014, Ni Ainle et al. 2009, Mittermayr et al. 2005, Mittermayr et al. 2009, Schultinge et al. 2023), with no indication of a heparin effect in the present study (Table 3).

While FXII depletion is not expected to impair hemostasis in vivo, its occurrence may still have practical implications for anticoagulation and hemostatic management. FXII deficiency can potentially cause misleading prolongation of clotting times in vitro which could affect interpretation of aPTT-based anticoagulation monitoring. This has been illustrated by a recent case report describing an unexpectedly prolonged baseline ACT during cardiac surgery due to FXII deficiency, which posed a diagnostic and management challenge (Apostel et al. ). Functionally driven tests such as viscoelastic assays may offer a more comprehensive assessment of coagulation dynamics and could help distinguish true hypocoagulability from assay-related artefacts. Further studies are needed to determine whether routine FXII measurement can contribute to individualized perioperative coagulation management.

### Influence of protamine

The reversal of heparin with protamine at the end of CPB represents an additional confounding factor. Protamine can transiently interfere with phospholipid-dependent coagulation assays, potentially masking factor-specific effects on the aPTT. In particular, high-dose protamine – as used in this study following a 1:1 regimen with heparin – was described to reduce the rate of FV activation and subsequent thrombin generation associated with a dose-dependent prolongation of the aPTT (Ni Ainle et al. 2009). Accordingly, the observed post-CPB decrease in FV activity may reflect a combination of dilutional or consumptive effects related to CPB and potential protamine-mediated inhibition. Moreover, early blood sampling three minutes after protamine administration could have further contributed to these measurements. Taken together, these factors underscore the need for cautious interpretation of post-CPB coagulation assays, particularly in the immediate post-protamine period. Future studies should specifically investigate the relative impact of protamine dose and sampling timing on intrinsic pathway assays to better differentiate true factor deficiency from assay-related interference.

### Comparison with ECMO data

Comparable findings have been reported in extracorporeal membrane oxygenation (ECMO), which also exposes blood to large artificial surfaces (Buchtele et al. 2021). In adults and children receiving ECMO, FXII deficiency is relatively common, and its dynamic decrease during extracorporeal circulation may affect anticoagulation monitoring (Brock et al. 2024, Drop et al. 2023). FXII activity declines markedly during ECMO (Buchtele et al. 2021), although baseline depletion and longer exposure time may influence the relative change compared with CPB in this study. Moreover, there are many differences between circuits, prime volumes, pumps and suckers, and reservoir, that could help explain why these two groups exhibit differences in coagulation. The rapid reduction of FXII during short-term CPB underscores the dynamic activation of the contact pathway and the need for precise coagulation management during extracorporeal circulation.

### Contact activation beyond coagulation

Beyond coagulation, the contact system contributes to inflammatory processes via activation of complement, neutrophils, and the bradykinin pathway (Veen et al. 2009, Maas and Renné 2018, Schmaier and McCrae 2007, Long et al. 2016). Recent work highlights that FXII and other contact activation system components directly link the intrinsic pathway with inflammatory signaling, including neutrophil activation and kallikrein-kinin-mediated responses, thereby reinforcing the immunothrombotic interplay during systemic inflammatory states (Wang and Zhu 2025). CPB triggers this activation promoting a systemic inflammatory response. The concomitant increase in FVIII observed in this study likely reflects its acute-phase nature as it is well described in cardiac surgery (Begbie et al. 2000, Jones et al. 1988, Ternström et al. 2010). Although FVIII activity tended to increase, this change did not reach statistical significance, which may be related to the small sample size in this study; previous studies have reported a significant increase in FVIII activity during CPB (Ternström et al. 2010). The complex interplay between coagulation and inflammation during CPB warrants further mechanistic investigation.

### Differences between surgical subgroups

The association between FXII activity and aPTT appeared to be cohort-dependent in our study. While only a weak correlation between FXII reduction and aPTT prolongation was observed in the aortic group (*ρ* = -0.312), the CABG patients demonstrated a strong, significant relationship (*ρ* = -0.835). This discrepancy suggests that the relationship between FXII activity and aPTT is not uniform across different surgical populations and is likely modulated by procedure-specific intraoperative factors. A similar context dependency may also apply to viscoelastic parameters, as reflected by the lack of correlation between HEPTEM CT and FXII activity. The observed discrepancy between the CABG and aortic cohort may be explained by several procedure-related mechanisms. First, in line with a masking-hypothesis, the more complex coagulopathic milieu in aortic surgery – characterized by hemodilution, factor consumption, enhanced fibrinolysis, and systemic inflammatory responses (Peterss et al. 2017) – may superimpose multiple, partly opposing effects on clotting time, thereby obscuring the isolated contribution of FXII to aPTT. In contrast, CABG causes less profound systemic perturbation, potentially allowing a more direct reflection of intrinsic pathway factor activity such as FXII on aPTT measurement. Second, a more pronounced inflammatory response in the aortic cohort may lead to stronger acute-phase increase in FVIII, which is known to shorten aPTT and could counterbalance or mask the prolongation expected from reduced FXII activity. Third, the deeper and more prolonged hypothermia typically employed in aortic procedures may alter coagulation enzyme kinetics in vivo, while laboratory-based aPTT and ROTEM measurements are performed under standardized conditions at 37 °C. This mismatch may result in a partial dissociation between functional FXII activity in the patient and its reflection in vitro, further weakening the observed association. Taken together, these considerations suggest that the relationship between FXII activity and coagulation measurements is context-dependent and may only be reliably interpretable under conditions of limited systemic perturbation, as more closely approximated in CABG surgery.

Despite comparable changes in coagulation factor activities across both cohorts following CPB, the extent of HEPTEM CT prolongation differed markedly, suggesting that viscoelastic CT may be influenced by additional procedure-related mechanisms beyond the measured coagulation factors. The generation of fibrinogen/fibrin split products may be of relevance as they have been shown to interfere with fibrin polymerization and clot formation, specifically in cardiac surgery (Belitser et al. 1982, Rifón et al. 1990, Gielen et al. 2015). Notably, higher concentrations of these may be expected in aortic surgery due to more extensive tissue injury. Additionally, methodological factors such as the mode of protamine administration and the timing of post-protamine blood sampling may have contributed to the observed differences with the aortic cohort potentially being more prone to protamine overdose due to increased heparin metabolism during CPB.

Importantly, this study was not designed to investigate the mechanistic basis of these differences, no dedicated analyses were performed to disentangle the relative contribution of the aforementioned factors. The observed divergence between the CABG and aortic cohort should therefore be interpreted as a hypothesis-generating finding. Future studies are warranted to systematically evaluate these mechanisms and to further clarify why the surgical groups behaved so differently, as well as to determine the perioperative conditions under which coagulation parameters and global assays reliably reflect coagulation factor activity.

### Limitations

This study included inherent limitations of such. It focused on a small, well-defined cohort of patients undergoing elective cardiac surgery, which may limit generalizability. Given the relatively small sample size, particularly within the subgroups, this may limit the statistical power to detect significant correlations between FXII and aPTT or HEPTEM CT. Generally, the high protamine-to-heparin ratio applied in this study may have influenced the measured coagulation parameters. Inflammatory and endothelial activation markers were not measured, although they could provide further insights into the interplay between coagulation and inflammation. Future studies should focus on the differences between CABG and aortic surgery in a lager patient population and include a broader set of variables such as fibrinogen/fibrin split products which may interfere with aPTT and viscoelastic measurements. Furthermore, future studies should incorporate postoperative follow-up and patient-centered clinical endpoints to assess the clinical implications of CPB-induced contact activation.

## Conclusions

This study demonstrates a significant CPB-induced decline in FXII activity. Global coagulation assays such as aPTT or ROTEM have limited sensitivity for isolated contact factor deficiencies; therefore, understanding assay-specific properties is essential for optimizing anticoagulation management during and after CPB. Acquired FXII reduction after CPB represents a diagnostic and interpretative issue rather than a clinically relevant bleeding risk. From a clinical perspective, our findings emphasize that in the absence of active bleeding and with preserved clot firmness in viscoelastic testing, isolated mild prolongation of intrinsic pathway assays after CPB requires cautious interpretation and should not routinely prompt escalation of hemostatic therapy.

## Data Availability

All data generated or analyzed during this study are included in this published article.

## Abbreviations

aPTT: Activated Partial Thromboplastin Time
A5/A20: Clot amplitude 5/20 min after Clotting Time
CABG: Coronary Artery Bypass Graft
CFT: Clot Formation Time
CPB: Cardiopulmonary Bypass
CT: Clotting Time
ECMO: Extracorporeal Membrane Oxygenation
FV: Factor V
FVIII: Factor VIII
FXII: Factor XII
INR: International Normalized Ratio
IQR: Interquartile Range
MCF: Maximum Clot Formation
